# Beta Atomic Contacts: Identifying Critical Specific Contacts in Protein Binding Interfaces

**DOI:** 10.1371/journal.pone.0059737

**Published:** 2013-04-22

**Authors:** Qian Liu, Chee Keong Kwoh, Steven C. H. Hoi

**Affiliations:** BIRC, School of Computer Engineering, Nanyang Technological University, Singapore, Singapore

## Abstract

Specific binding between proteins plays a crucial role in molecular functions and biological processes. Protein binding interfaces and their atomic contacts are typically defined by simple criteria, such as distance-based definitions that only use some threshold of spatial distance in previous studies. These definitions neglect the nearby atomic organization of contact atoms, and thus detect predominant contacts which are interrupted by other atoms. It is questionable whether such kinds of interrupted contacts are as important as other contacts in protein binding. To tackle this challenge, we propose a new definition called beta (*β*) atomic contacts. Our definition, founded on the *β*-skeletons in computational geometry, requires that there is no other atom in the contact spheres defined by two contact atoms; this sphere is similar to the van der Waals spheres of atoms. The statistical analysis on a large dataset shows that *β* contacts are only a small fraction of conventional distance-based contacts. To empirically quantify the importance of *β* contacts, we design *β*ACV, an SVM classifier with *β* contacts as input, to classify homodimers from crystal packing. We found that our *β*ACV is able to achieve the state-of-the-art classification performance superior to SVM classifiers with distance-based contacts as input. Our *β*ACV also outperforms several existing methods when being evaluated on several datasets in previous works. The promising empirical performance suggests that *β* contacts can truly identify critical specific contacts in protein binding interfaces. *β* contacts thus provide a new model for more precise description of atomic organization in protein quaternary structures than distance-based contacts.

## Introduction

Specific binding between proteins plays a fundamental role in molecular functions and biological processes. The discovery of governing principles behind specific protein interactions is thus an essential issue in proteomics. Protein interactions are generally considered to be governed by their binding interfaces which consist of interfacial residues/atoms and their contacts. In order to uncover contributing factors to specific interactions, binding interfaces and their contacts are firstly quantified according to several widely-used criteria: some definitions consider atomic distance between atoms each from one protein [1]–[7], while another more complicated definition takes into account Voronoi diagrams of entire complexes [8]–[13]; the other criterion defines binding interfaces using the change of solvent accessible surface area (ΔASA) upon the formation of protein complexes [14]–[21]. Under these definitions, protein binding is found to be driven by forces from those atomic contacts such as hydrogen bonds, electrostatic interactions, van der Waals forces, salt bridges, hydrophobic attractions, etc.

However, these criteria define an interface simply as a cluster of spatially close atoms and their contacts but pay little attention to the local surroundings of its defined contacts. Thus, a lot of non-specific contacts are detected, which makes it still very difficult, if not completely impossible, to pinpoint the governing principles according to these existing contact definitions. One piece of evidence for the non-specific contacts is that these definitions will detect larger ‘binding interfaces’ in crystal packing, and it is very hard to distinguish these crystal-packing ‘binding interfaces’ from true ones. Here, crystal packing is the artifact of the crystallographic packing environments and is randomly formed during the crystallization process; but they do not occur in solution or in their physiological states [22]. With crystal packing as the reference state, a perfect contact definition is expected to satisfy that no or fewer contacts are detected in crystal packing; based on this definition, crystal packing should be easily distinguished from specific biological binding of proteins using a simple learning algorithm.

In this work, we propose a new definition: *β* atomic contacts. A *β* atomic contact *β* of atoms *β* and *j* must satisfy *β*-skeletons [23] where *c*'s forbidden region contains no other atom. This forbidden region is defined by the parameter *β*. In this work, *β* is set to 1, defining a sphere with the midpoint of *i* and *j* as the center and with the spatial distance between *i* and *j* as the diameter (similar to the van der Waals spheres of atoms). Thus, our definition only detects “perceptually meaningful” contacts. We expect *β* atomic contacts to provide a more precise model of atomic organization in protein 3D structures than the previous definitions.

To demonstrate the efficacy of our *β* contacts in identifying critical atomic contacts in protein binding interfaces, we adopt *β* atomic contacts to define protein interfaces and then investigate the difference between homodimeric interfaces and crystal-packing interfaces. Many previous works also endeavored to detect distinguishing characteristics of crystal packing and specific biological binding. Some works have revealed a significant difference between protein surfaces and interfaces in amino acid composition, as well as a high similarity of protein surfaces to crystal packing [16], [18], [24]–[26]. Several other methods have been proposed to identify biological protein complexes from crystal packing. Both Weng's group [27] and Klebe's group [28] represented interfaces using atomic contact vectors (ACV), and then took them as inputs of machine-learning algorithms to construct efficient classifiers for distinguishing different types of protein binding, such as permanent and transient interactions and crystal packing [27], [28]. PITA scored crystal packing using their contact size and chemical complementarity [29]. Zhu *et al.* [15] extracted six properties from interfaces, such as interface size, amino acid composition and gap volume, and then fed them into an SVM to train their NOXclass classifier to discriminate obligate and non-obligate interactions and crystal packing [15]. Using residue-based Voronoi tessellations of protein structures, Bernauer *et al.* constructed an SVM classifier DiMoVo for identifying biological protein interactions [11]. Taking the advantage of the hypothesis that energetically important residues are generally protected by the O-ring [30], Liu and Li designed the propensity vector of residue contacts within the O-ring to develop OringPV for the distinction between crystal packing and biological interactions and between two different types of biological interactions [31]. However, almost all of them use knowledge extracted from the simple definitions, such as defining interfaces using ASA change or defining interfacial contacts using a threshold of atomic distance.

In this work, we use *β* atomic contacts in interfaces to classify homodimers from crystal packing. In this classification, we represent an interface by an ACV [27] based on *β* contacts, and then design a new classifier, called *β* atomic contact vector (*β*ACV). *β*ACV is a linear SVM classifier with selected distinguishable types of *β* atomic contacts by SVM-RFE as input. Evaluated on several previous datasets, *β*ACV achieves better classification than the ACV classifier simply based on the distance-based contacts, although *β* atomic contacts are only a small fraction of the contacts under the latter definition. Our *β*ACV is also compared with several existing methods in the literature, including PISA, DiMoVo and NOXclass. The results demonstrate that *β* contacts are superior to these methods in most cases. All these comparisons suggest that *β* contacts are more capable of capturing specific binding contacts than the other definitions. A web server of the proposed *β*ACV solution is also available at http://sunim1.sce.ntu.edu.sg/liuqian/bacv/index.py.

## Materials and Methods

### Datasets

Three datasets in the literature are used to comprehensively evaluate *β* atomic contacts.

The first *Bahadur* dataset contains 178 crystal packing and 113 biological homodimers from the previous works [16], [19]. This dataset has been used to develop DiMoVo [11].

The second *Ponstingl* dataset has 95 crystal packing and 76 homodimers [32]. This dataset has been used in several existing works [27], [28], including PITA [29] and PISA [33].

The third *non-redundant* dataset is compiled by [11] from the Bahadur, Ponstingl and NOXclass datasets [15]. In this non-redundant dataset, two proteins have no more than 30% sequence identity. This dataset includes 314 crystal packing and 144 homodimers after preprocessing [11].

### What are β atomic contacts

As presented in the File S1, the various definitions of atomic contacts proposed in previous works have several limitations. To tackle these limitations, we propose a new definition–-*β* atomic contacts. Given a protein complex *p*, **an atomic contact, denoted as** *c*(*i*,*j*)**, between two atoms** *i* **and** *j* **is called a** *β* **contact if and only if** 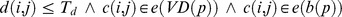. Specifically, these three requirements are described as follows:


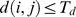
: This first requirement states that the surface distance between *i* and *j*, *d*(*i*,*j*), must be less than or equal to 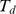. Here, a surface distance of two heavy atoms is their Euclidian distance minus the sum of their van der Waals radii as defined in [34]. A similar surface distance definition is also used in [5], [35], [36]. For a simple description, the ‘distance’ in our definition and method is always calculated in this way except when otherwise specified. These contacts under this requirement are called distance-based contacts.


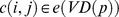
: For the second requirement, *i* and *j* must share a Voronoi facet in 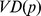 where 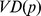 is the Voronoi diagram of *p* and 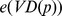 is a set of edges in 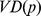. These contacts under the two requirements above are called Voronoi-based contacts.


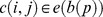
: The last requirement indicates that 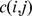 cannot break *β*-skeletons [23]. That is, 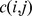 is an edge of the *β*-skeleton *b*(*p*) of *p* where 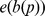 is a set of edges in *b*(*p*). These contacts under the three requirements above are called *β* atomic contacts.

**Figure 1:**
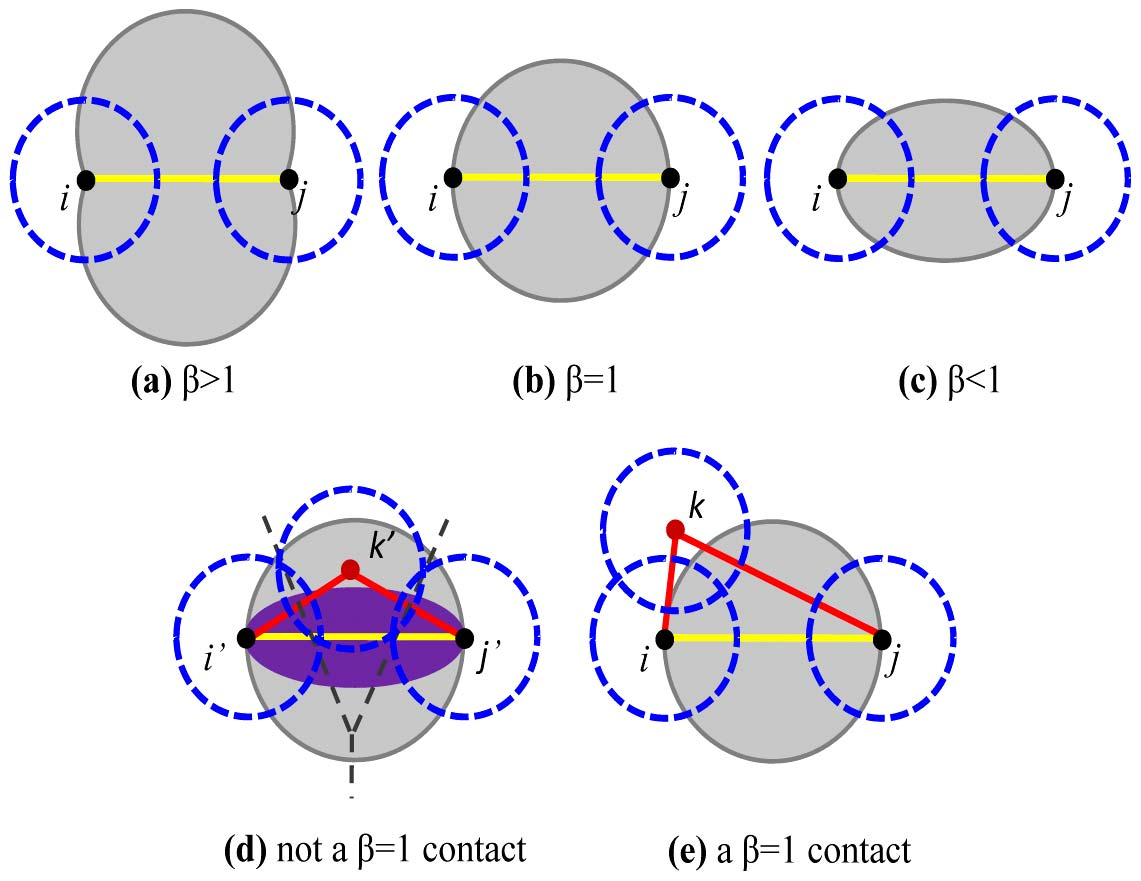
*β* **skeletons and** *β* **contacts.** Three points, *i*, *j* and *k*, represent the atoms. The dash circles in blue represent van der Waals spheres in 2D space. The lines in yellow are of interest. In the first row, if *i* and *j* have a *β* contact, their surface distance is less than a threshold 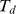 and the gray regions are required to contain no other atom by *β* skeletons when *β* > 1 (left), *β*  =  1 (center) and *β* < 1 (right), respectively. In (d), a region in magenta is the center area which is very close around the line in yellow, and the dash lines represent Voronoi facets.

A *β*-skeleton of a discrete set *p* is an undirected graph in computational geometry where two points *i* and *j* have an edge if any angle 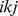 is sharper than a threshold determined by *β*,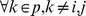. *β* actually defines a forbidden region for the contact between *i* and *j*, just like the gray regions in Figure 1(a)–(c) with different *β* values. In Figure 1, two atoms *i* and *j* have an edge in *β*-skeletons if there are no other atoms *k* whose center is in their forbidden region. In other words, if there is any atom whose center is in the gray region, the atomic contact between *i* and *j* is interrupted, and the contact should not exist in *β*-skeletons. For example when *β*  =  1, two atoms *i*' and *j'* do not have a *β* contact in Figure 1(d) because there is a *k*' in their forbidden region, while the contact between two atoms *i* and *j* is a *β* contact in Figure 1(e) since any atom *k* is outside the forbidden region. It is also interesting to note that in Figure 1(d), two atoms *i*' and *j'* have a Voronoi-based contact, but they only share a smaller-size facet (e.g., the dash gray line down in Figure 1(d)) which is also far away from the center region of the contact–-the center region of the contact between two atoms *i*' and *j'* is a small arch region very close around the contact, that is, the magenta region in Figure 1(d). Compared to this contact in the Voronoi-based definition, our *β* criterion assumes that two atoms should have enough contact area in their center region to form an important interaction.

In *β*-skeletons, with different values of *β* from bigger to smaller, the forbidden gray regions decrease as shown in Figure 1 from (a) to (c), and thus the number of atomic contacts in *β*-skeletons increases. When *β* is small enough, the contacts defined on *β*-skeletons are similar to those on Voronoi diagrams or even to those on distance-based definitions. In this work, *β* is set to 1, and this *β*-skeleton is also called the Gabriel graph [37], [38]. We would like to emphasize that (i) *β*-skeletons are a totally different concept from *β* shape [39], a generalization of the 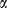 shape [40], which is also commonly used in the analysis of protein structures, such as ASA calculation and protein shape detection; and (ii) *β* contacts can be either long-range contacts or short-range contacts in tertiary structures which are different contact definitions based on both sequence separation and spatial distance(please refer to the references [41]–[43] for the definitions of long-range contacts); but short- and long-range contacts focus on sequence separation while *β* contacts emphasize the spatial organization of atomic interactions.

### Detecting β atomic contacts in protein 3D structures

A protein 3D structure can be modeled as a *β* atomic contact graph *b*(*p*) where heavy atoms are considered as points, and *β* atomic contacts as edges. Given a protein 3D structure, its *b*(*p*) can be produced by the following process.

First, Qhull is used to produce Delaunay triangulation [44] for all points. Second, a surface-distance threshold 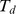 is used to remove those atomic contacts whose distances are too large. 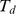 is set to 3.3 Å (the diameter of a water molecule 2.8 Å plus 0.5 Å). 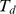 is the maximum surface-distance between the van der Waals spheres of two atoms, as discussed in the *β* contact definition. Thirdly, each atomic contact is checked to guarantee that it satisfies *β* skeletons, that is, the Gabriel graph here. Since we are interested in atomic contacts between proteins, all atoms which have no contact across binding interfaces are removed.

### *β* atomic contact vectors in protein interfaces

An atomic contact vector (ACV for short) [27] is adopted to represent an interface. In this vector representation, all heavy atoms of the twenty standard residues in proteins are grouped according to twelve atomic types in the File S1. These atomic types are similar to those in [45]. Hence, the atomic contact vector for an interface has 78 atomic pairs (78 = 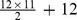). The value for each pair is its occurrence in a *β* atomic contact graph when 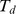 is set to 3.3 Å. Since disulfide bonds are almost as strong as covalent bonds, disulfide bonds whose spatial distance of two sulfur atoms across interfaces is less than 2.6 Å are also considered as an atomic pair in the vector. Finally, the vector for a protein interface has 79 pairs, called *β*ACV1a (*β* atomic contact vectors) for short. Similarly, we also construct *β*ACV1 in which the surface-distance threshold of two contact atoms is as small as 0.5 Å, that is, 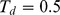 Å.

In addition to atomic types, the distance between contact atoms is also an important factor in protein binding. Given two atomic pairs with the same types, one pair has a small distance between atoms, while atoms in the other pair have a much larger distance; the first pair generally has different importance to protein binding from the second pair. One example with this property is hydrogen bonds in interfaces: if a Nitrogen atom and an Oxygen atom have less than 3.5 Å spatial distance, their contact may be a hydrogen bond; but these two atoms cannot form a hydrogen bond directly if their spatial distance is too large, for example, more than 5 Å. Therefore, we take into account the surface-distance information of atomic contacts and split *β*ACV1a into three sub-vectors: each of them contains atomic contacts whose surface-distance falls in one of the three regions: ≤0.5, (0.5,1.9] and (1.9,3.3], and they are named as ≤0.5 contacts, (0.5,1.9] contacts and (1.9,3.3] contacts for short. Here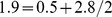. This vector representation has 235 pairs (235 = (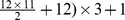), which is referred to as *β*ACV3 for short.

Meanwhile, to enable a fair comparison, distance-based ACV1 (dACV1), ACV1a (dACV1a) and ACV3 (dACV3) are also constructed in a similar way.

### Our proposed classifier *β*ACV and evaluation measures

We want to evaluate *β* contacts in classifying homodimers and crystal packing. In our classification task, a dataset of crystal packing and homodimers is represented by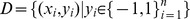, where 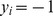 indicates that this vector is from crystal packing or 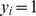 indicates it is from homodimers; *n* is the total number of crystal packing and homodimers; 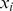 is the *β* atomic contact vector (*β*ACV1, *β*ACV1a or *β*ACV3) for interface *i*. SVM with a linear kernel in LIBSVM [46] (the freely available SVM library) is then employed to train our classifier for identifying homodimers from crystal packing. A short description of SVM is provided in the File S1.

In our *β*ACV classifier, SVM-RFE (a short description of RFE is provided in the File S1) is firstly used to find the feature set 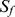 with the best accuracy for the features *β*ACV1 or *β*ACV1a or *β*ACV3. SVM-RFE uses SVM learning to obtain feature weight 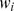 and then removes features with the lowest value 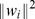 step-by-step until the predefined criteria are satisfied. This process uses a five-fold cross-validation. Then, two established ways are used to evaluate classification performance. One is feature-selection classification performance by using a leave-one-out cross-validation on the learning datasets. The other is the independent-dataset testing. That is, a *β*ACV classifier with features 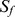 is constructed for predicting those complexes whose proteins have low sequence similarity to the complexes in the dataset for feature selection. The independent-testing datasets for the Bahadur, Ponstingl and NOXclass datasets can be found in [11].

Finally, 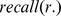, 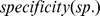, 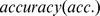, and Matthew's correlation coefficient 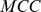 are adopted to evaluate the classification performance of *β* atomic contact vectors. Their definitions are provided in the File S1. MCC is more meaningful in a dataset which has a significant imbalance between the numbers of positive and negative samples.

## Results and Discussion

### Comparison of *β* atomic contacts with distance-based atomic contacts

To demonstrate that *β* contacts are better than distance-based contacts in describing protein binding interfaces, we compare these two types of definitions from the following aspects. We firstly measure the numbers of distance-based contacts and *β* atomic contacts to see their difference. We then compare their prediction performance on the three datasets. Following that, we provide a detailed comparative analysis of the selected features by RFE, especially of the top 10 atomic-contact features, for distance-based contacts and *β* contacts.

#### *β* atomic contacts are a small fraction of distance-based atomic contacts

We perform statistical analysis of the number of atomic contacts under the three different definitions, distance-based, Voronoi-based, and *β* contacts. The analysis is based on the non-redundant dataset where 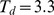. The result is shown in Table 1.

**Table 1:** The difference of the numbers of distance-based, Voronoi-based and *β* atomic contacts for 114 homodimers and 314 crystal packing.

	in homodimers			in crystal packing		
	Distance-based	Voronoi-based	*β* contacts	Distance-based	Voronoi-based	*β* contacts
Voronoi-based	*508,792*	0	*71,126*	*265,293*	0	*49,425*
*β* contacts	*579,918*	*71,126*	0	*314,718*	*49,425*	0
Number of contacts	**647,878**	**139,086**	**67,960**	**354,652**	**89,359**	**39,934**
	(4,499.2±2,822.3)	(965.9±572.8	(471.9±286.4)	(1,129.5±533.7)	(284.6±125.0)	(127.2±57.2)

The numbers in *Italics* are the difference of the numbers of the contacts under the definitions of its column and row.

The number of the last two rows in **bold** is the number of atomic contacts under the definition of its column.

X±Y in last row: X is the mean of the number of atomic contacts in interfaces of a dataset while Y is the standard deviation.

From Table 1, we find that *β* atomic contacts are only a small fraction of distance-based atomic contacts, i.e., 10.5% of distance-based contacts in homodimers, and 11.3% in crystal packing.

**Figure 2:**
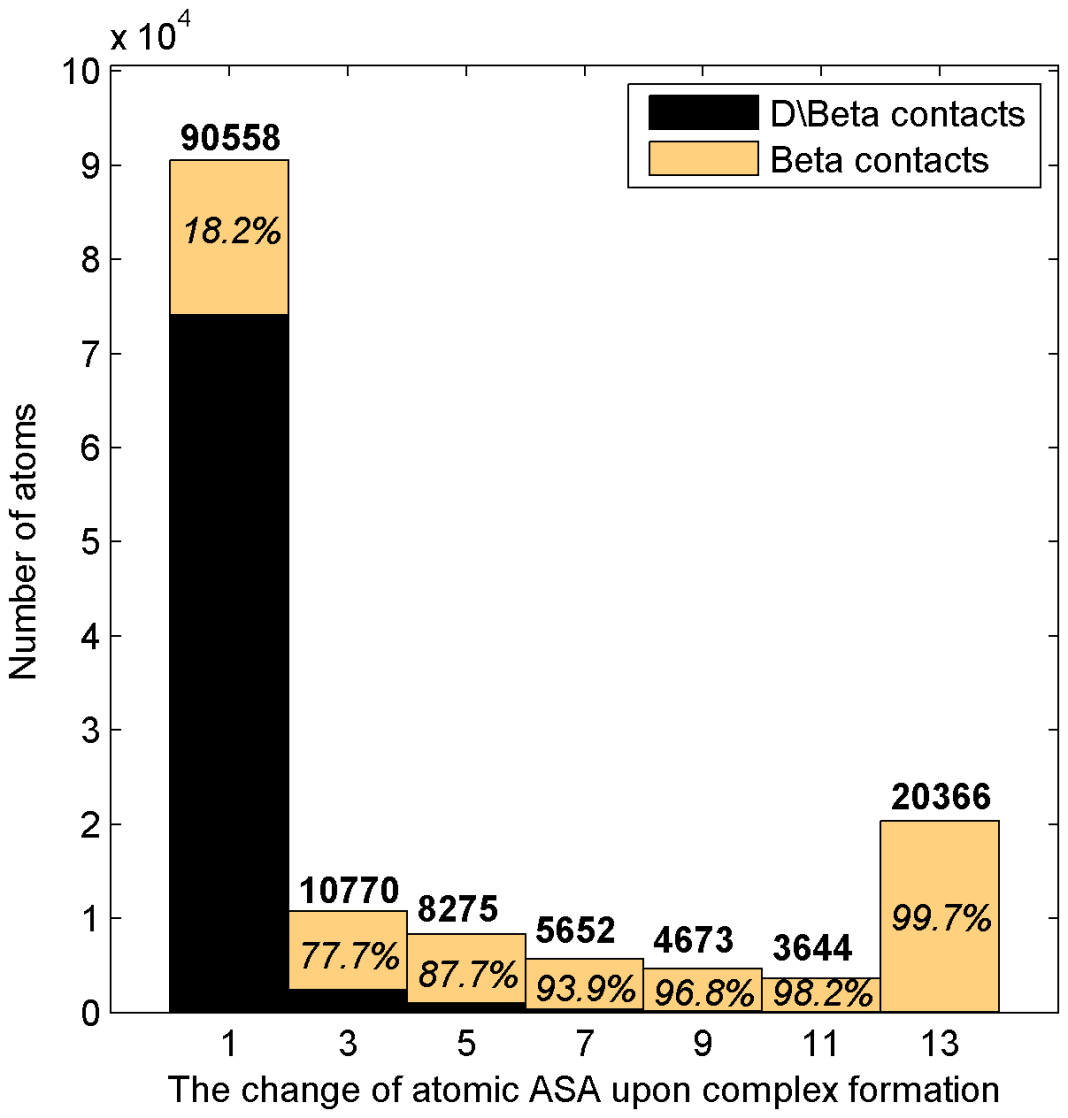
The ASA change (Å) of atoms in *β* **contacts and in the complement of** *β* **contacts with respect to distance-based contacts (referred to as ‘D** **\Beta contacts’ for short in the figure).** The integer in bold on a bar is the number of atoms whose ASA change falls in the region of the bar, while the percent in *italics* is the corresponding percentage of atoms only in *β* contacts.

We also show the comparison of the atoms involved in *β* contacts with those in the complement of *β* contacts with respect to distance-based contacts in Figure 2. These atoms and their ASA are from the non-redundant dataset with 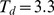. It seems that a lot of contact atoms in distance-based contacts are not defined to be in direct interfaces according to *β* contacts; Figure 2 also clearly shows that atoms only in distance-based contacts mostly have small ASA change after complex formation, while the atoms with larger ASA change are mostly in *β* contacts.

#### *β* atomic contacts and distance-based atomic contacts in the classification of crystal packing and homodimers

We then compare *β* contacts with distance-based contacts in classifying crystal packing and homodimers. The results are shown in Tables 2 and 3.

**Table 2:** The comparison of feature-selection classification performance achieved by distance-based and *β* atomic contacts and other methods in the literature.

Dataset		distance-based contacts			*β* contacts			DiMoVo	Ref [28]
		dACV1	dACV3	dACV1a	*β* ACV1	*β* ACV3	*β* ACV1a		
Ponstingl	r.	0.895	0.895	0.908	0.895	0.934	0.921	-	-
	sp.	0.947	0.947	0.947	0.937	0.968	0.989	-	-
	acc.	**0.924**	0.924	0.93	0.918	**0.953**	**0.959**	-	0.948
	MCC	**0.846**	0.846	0.858	0.834	**0.905**	**0.918**	-	-
Bahadur	r.	0.840	0.877	0.821	0.887	0.925	0.877	0.890	-
	sp.	0.955	0.955	0.955	0.983	0.983	0.943	0.980	-
	acc.	0.911	0.926	0.904	**0.947**	**0.961**	**0.918**	0.945	-
	MCC	0.810	0.840	0.795	**0.887**	**0.917**	**0.825**	0.884	-
Nonredundant	r.	0.882	0.917	0.861	0.896	0.958	0.868	-	-
	sp.	0.978	0.971	0.978	0.984	0.994	0.975	-	-
	acc.	0.948	0.954	0.941	**0.956**	**0.983**	0.941	-	-
	MCC	0.877	0.893	0.862	**0.898**	**0.959**	0.862	-	-

r., sp., acc. and MCC represent recall, specificity, accuracy, and Matthew's correlation coefficient respectively. The **bold numbers** are the larger values in each of three pair-wise comparisons of *β*ACV1 vs dACV1, *β*ACV3 vs dACV3, and *β*ACV1a vs dACV1a. In this table, significant features are selected on a dataset, and then a method with the selected features is evaluated on this dataset under a leave-one-out cross-validation process, as discussed in **Materials and Methods**.

**Table 3:** The comparison of classification performance on the independent datasets achieved by distance-based and *β* atomic contacts and other methods in the literature.

Dataset		distance-based contacts			*β* contacts			*D*\*β* contacts^1^		DiMoVo	PISA	PITA	NOXclass
Training on		dACV1	dACV3	dACV1a	*β* ACV1	*β* ACV3	*β* ACV1a	*D*\*β* ACV1^1^	*D*\*β* ACV3^1^				
Ponstingl	r.	0.908	0.934	0.934	0.947	0.947	0.921	0.803	0.908	0.710	0.920	0.840	-
	sp.	0.934	0.929	0.898	0.938	0.934	0.938	0.863	0.929	0.920	0.760	0.910	-
	acc.	0.927	0.930	0.907	**0.940**	**0.937**	**0.934**	0.848	0.924	0.868	0.802	0.894	-
	MCC	0.815	0.827	0.780	**0.851**	**0.844**	**0.832**	0.627	0.808	0.643	0.602	0.730	-
Bahadur	r.	0.868	0.974	0.947	0.842	0.947	0.974	0.816	0.921	0.840	-	-	-
	sp.	0.986	0.978	0.986	1.000	0.971	0.971	1.000	0.986	0.950	-	-	-
	acc.	0.960	**0.977**	**0.977**	**0.966**	0.966	0.972	0.960	0.972	0.929	-	-	-
	MCC	0.880	**0.935**	**0.933**	**0.898**	0.902	0.920	0.881	0.915	0.780	-	-	-
NOXclass	r.	0.861	0.924	0.937	0.911	0.937	0.810	0.759	0.924	0.790	-	-	0.950
	sp.	0.909	0.841	0.868	0.909	0.882	0.877	0.714	0.836	0.970	-	-	0.680
	acc.	0.896	0.863	**0.886**	**0.910**	**0.896**	0.860	0.726	0.860	0.920	-	-	0.751
	MCC	0.745	0.702	**0.747**	**0.784**	**0.765**	0.659	0.424	0.697	0.790	-	-	0.556

r., sp., acc. and MCC represent recall, specificity, accuracy, and Matthew's correlation coefficient respectively. The **bold numbers** have the same meaning as those in Table 2. Here, significant features and a prediction method on them are trained on a dataset, and the evaluation is performed on another dataset, as discussed in **Materials and Methods**. ^1^ *D*\*β* contacts are the complement of *β* contacts with respect to distance-based contacts, while *D*\*β*ACV1 and *D*\*β*ACV3 are ACV vectors based on *D*\*β* contacts.

It is clearly seen from Table 2 that *β* contacts aggregately have better performance than distance-based contacts, although *β* contacts are only a small fraction of distance-based contacts. In the nine pair-wise comparisons in Table 2 (three pairs of *β*ACV1 versus dACV1, *β*ACV3 versus dACV3, and *β*ACV1a versus dACV1a on the three datasets), *β* contacts are superior in seven times, and distance-based contacts are superior in only one (there is one tie). For example on Bahadur, *β*-contact *β*ACV3 has much better MCC, with 7.7 percent points higher than distance-based dACV3.

Table 3 presents the classification performance of *β* contacts and distance-based contacts on the independent datasets. In these nine comparisons, *β* contacts perform better in six times. Again, *β* contacts aggregately demonstrate better classification performance than distance-based contacts.

The difference between *β* contacts and distance-based contacts are also evaluated by *D*\*β*ACV1 and *D*\*β*ACV3 which only use the corresponding complement of *β* contacts with respect to distance-based contacts, that is, those atomic contacts not in *β*ACV1 and *β*ACV3 but in dACV1 and dACV3, respectively. The results are shown in Table 3. *β* contacts still achieve better performance on the independent datasets of NOXclass and Ponstingl and similar performance on the independent dataset of Bahadur. This similar performance should be due to the fact that this independent dataset is easily distinguished with high classification performance for both distance-based and *β* contacts (as shown in Table 3). Meanwhile, *D*\*β*ACV3 has similar performance to dACV3, because the distance-based contacts predominate in their used contacts.

From Tables 2 and 3, we observe that (i) *β*ACV1 can achieve good classification performance, which suggests that atomic contacts with smaller distance play a vital role in protein binding; (ii) *β*ACV3 aggregately outperforms *β*ACV1 and *β*ACV1a, indicating that atomic contacts with relatively larger distance can also contribute to protein binding and atomic contacts with different distances have various contributions to protein interactions. In our web server, *β*ACV3 has been made available to the scientific community.

In conclusion, *β* contacts are generally more capable in capturing critical specific binding contacts than distance-based contacts.

### Analysis of the selected features in the classifications

#### Top 10 selected features in *β* atomic contacts and in distance-based atomic contacts

To deepen our understanding of the difference between *β* atomic contacts and distance-based atomic contacts, we show the top 10 selected features of *β*ACV3 and dACV3 by SVM-RFE in Table 4 when *β*ACV3 and dACV3 are trained on the non-redundant dataset.

**Table 4:** The top 10 features of distance-based and *β* atomic contacts when *β*ACV3 and dACV3 are trained on the non-redundant dataset.

Rank	distance-based contacts		*β* contacts	
	types of atomic contacts	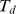 range	types of atomic contacts	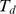 range
1st	N_3_H_1__ **C_4_H_3_**	(1.9,3.3]	**C_3_ (S_2_)H_0_**___ **C_4_H_3_**	≤0.5
2rd	**C_4_H_1_**___ **C_4_H_1_**	(1.9,3.3]	N_3_H_2__ **C_3_ (S_2_)H_1_**	≤0.5
3th	*O*_2_ *H*_1__ **C_4_H_2_**	(1.9,3.3]	**C_4_H_1_**___ **C_4_H_3_**	≤0.5
4th	N_3_H_1__ *O*_1_ *H*_0_ *-*	(1.9,3.3]	*O*_1_ *H*_0_ *-*___ *O*_2_ *H*_1_	(1.9,3.3]
5th	**C_3_ (S_2_)H_0_**___ **C_3_ (S_2_)H_1_**	(1.9,3.3]	N_4_H_3_/2+___ **C_4_H_1_**	(1.9,3.3]
6th	N_3_H_1__ **C_4_H_1_**	(1.9,3.3]	*O*_1_ *H*_0_ *-*___ *O*_1_ *H*_0_ *-*	(1.9,3.3]
7th	*O*_1_ *H*_0__ **C_4_H_3_**	(1.9,3.3]	*O*_2_ *H*_1__ **C_4_H_2_**	≤0.5
8th	*O*_1_ *H*_0__ *O*_1_ *H*_0_	(1.9,3.3]	N_3_H_1__ *O*_1_ *H*_0_	≤0.5
9th	**C_3_ (S_2_)H_1_**___ **C_3_ (S_2_)H_1_**	(0.5,1.9]	**C_4_H_3_**___ **C_4_H_3_**	(1.9,3.3]
10th	**C_4_H_3_**___ **C_4_H_3_**	(1.9,3.3]	**C_4_H_1_**___ **C_4_H_1_**	(0.5,1.9]

X_Y means atomic contacts between X and Y, while X and Y are atomic types in the File S1. Carbon atoms are in **bold**, while Oxygen atoms are in *italics*.

From Table 4, these top 10 features of *β* atomic contacts indicate two interesting phenomena. One is the hydrophobic effect–-the contacts among Carbon atoms are chosen to be significantly important to biological binding, although their atomic types are different and their surface distances are ≤0.5 Å, or in (0.5,1.9] Å, or in (0.5,1.9] Å. The other is that hydrogen bonds, ≤0.5 contacts between N_3_H_1_ and O_1_H_0_ in *β* contacts, also play an important role in classifying biological binding from crystal packing.

The top 10 selected features of distance-based contacts capture contacts among Carbon atoms, but miss hydrogen bonds. Furthermore, most of the top features of distance-based contacts are those atomic contacts with relatively larger surface-distance, in (1.9,3.3] Å in Table 4. The main reason is the spatial constraint: given a sphere with an atom as the center, the larger the radius is, i.e., the larger surface-distance threshold 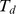 here, the more other atoms can be covered without atomic clashes. That is, in distance-based contacts, the number of contacts with the bigger surface-distance (1.9,3.3] Å is generally much larger than the number of contacts with the smaller surface-distance ≤0.5 Å. However, this does not hold in *β* contacts. For example on the non-redundant dataset, the number of ≤0.5 contacts in *β* contacts is 43,063, and the number of (1.9,3.3] contacts is 14,007. (1.9,3.3] contacts are about one-third of ≤0.5 contacts in *β* contacts. However in distance-based contacts, the number of ≤0.5 contacts and that of (1.9,3.3] contacts are 57,286 and 635,254; (1.9,3.3] contacts are over ten times more than ≤0.5 contacts in distance-based contacts. This misleads SVM and RFE to prefer the (1.9,3.3] contacts in distance-based contacts, since they have a higher occurrence.

With the discussion above in mind, one argument in distance-based contacts is: when the (1.9,3.3] contacts mislead SVM and RFE, why the SVM classifier does not have much worse performance. There are at least two helpful factors contributing to classifiers based on distance-based contacts. One is that distance-based contacts can easily represent atomic density in interfaces; the other is that interface contact size can also greatly help the classification performance of distance-based contacts. Both atomic density and contact size should be distinguishable features and a possibly necessary condition for biological binding; they are easily but indirectly implied in distance-based ACV3 vector, although the contacts are divided into different types. However, both of them should not be sufficient conditions for specific protein binding.

#### Decision trees of distinguishing features in *β* atomic contacts and in distance-based atomic contacts

**Figure 3:**
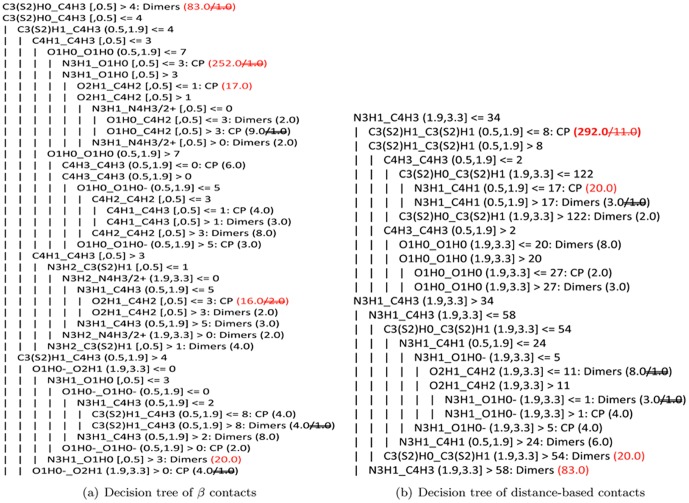
Decision tree of *β* **contacts on the non-redundant dataset.** Each line is a branch in decision trees; ‘
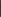
’ and an indent represent a sub-branch; ‘Dimers’ indicates a class label of biological binding, while ‘CP’ refers to a class label of crystal packing; means the number of misclassified complexes in a branch; the format of a line is: types of atomic contacts with their surface-distance information, followed by a splitting rule and a class label if possible. For example, the rule ‘C_3_ (S_2_)H_0__C_4_H_3_ [,0.5] >4: Dimers (83.0)’ in the first line of Figure 3(a) suggests the following prediction: 83 interfaces have more than four ≤0.5 contacts of C_3_ (S_2_)H_0_ and C_4_H_3_ in the non-redundant dataset; among these interfaces, only one is crystal packing.

To visualize the selected features by RFE and to provide some clues of governing principles underlying protein binding, we show the decision trees in Figure 3(a) for *β* contacts and in Figure 3(b) for distance-based contacts only using these selected features when *β*ACV3 and dACV3 are trained on the non-redundant dataset. The details of how to construct decision tree are provided in the File S1. With these selected features, *β*ACV3 and dACV3 achieve accuracy of 0.983 and 0.954; in the decision trees with 5-fold cross-validation, *β* contacts have accuracy of 0.891, and distance-based contacts have accuracy of 0.915. Since the important features in SVM cannot be guaranteed to be the same as those in the decision trees, we do not pay more attention to the similar performances of the two trees. Instead, we would like to see whether easily interpretable knowledge can be derived from these two decision trees, because SVM-based *β*ACV3 has much better classification performance but poor interpretability.

Figure 3(a) suggests three interesting rules. One is about contacts between two Carbon atoms, called R1 in the first line of Figure 3(a). R1 suggests that if an interface has more than four ≤0.5 contacts between the atomic types C_3_ (S_2_)H_0_ and C_4_H_3_ (*C*_3_ *(S*_2_ *)H*_0_ *_C*_4_ *H*_3_ for short), it has a probability of 98.8%(82/83) to be a biological binding. This rule is consistent with the hydrophobic effect. The other two interesting rules are closely related to hydrogen bonds. One hydrogen-bond-involving rule is: given an interface with less ≤0.5 C_3_ (S_2_)H_0__C_4_H_3_, it can still be biological binding if this interface has: **(i)** more than four (0.5,1.9] C_3_ (S_2_)H_1__C_4_H_3_ contacts, and **(ii)** more than three hydrogen bonds (≤0.5 N_3_H_1__O_1_H_0_ contacts), and **(iii)** no (1.9,3.3] O_1_H_0_-_O_2_H_1_ contacts. In the non-redundant dataset, 20 biological binding interfaces and none of the crystal packing satisfy this rule. In contrast, 251 crystal packing and only one biological interface satisfy the other hydrogen-bond-involving rule (called R2 for short). As shown in Figure 3(a), R2 requires an interface with **(i)** less than five ≤0.5 C_3_ (S_2_)H_0__C_4_H_3_ contacts, **(ii)** less than five (0.5,1.9] C_3_ (S_2_)H_1__C_4_H_3_ contacts, **(iii)** less than four ≤0.5 C_4_H_1__C_4_H_3_ contacts, **(iv)** less than eight (0.5,1.9] O_1_H_0__O_1_H_0_ contacts, and **(v)** not more than three hydrogen bonds (≤0.5 N_3_H_1__O_1_H_0_ contacts). This rule is a little more complicated, but it is reasonable: there should be more than one type of specific important contacts to protein binding, and enough occurrence of several of them in an interface should produce a true biological binding; hence, a false biological binding should not have enough critical specific contacts, which must exclude all potential combinations of specific important contacts. This makes a complicated rule unique to crystal packing.

According to these three rules of *β* contacts in Figure 3(a), we believe that the following contacts should be closely related to specific important contacts to protein binding: the ≤0.5 contacts of C_4_H_3_ with C_3_ (S_2_)H_0_ and with C_4_H_1_, (0.5,1.9] C_4_H_3__C_3_ (S_2_)H_1_ contacts, and ≤0.5 N_3_H_1__O_1_H_0_ contacts. These contacts are consistent with previous observations: ≤0.5 N_3_H_1__O_1_H_0_ contacts generally can be hydrogen bonds; C_4_H_3_ are mostly in the side chains of such hydrophobic residues as Val, Ile and Leu, indicating the hydrophobic effect; C_3_ (S_2_)H_1_ are almost in the aromatic side chains, providing 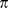-involving interactions in binding interfaces. In contrast, it is crystal packing, not biological binding, which prefer (1.9,3.3] O_1_H_0_-_O_2_H_1_ contacts. Further in Figure 3(a), higher occurrence of contacts involving O_1_H_0_- almost suggests crystal packing prediction. O_1_H_0_- should play a destructive role in binding interfaces unless it can form salt bridges.

Similarly, distance-based contacts in Figure 3(b) also suggest two interesting rules. One is that only 83 biological binding have more than fifty-eight (1.9,3.3] N_3_H_1__C_4_H_3_ contacts; the other is that in those interfaces which have not more than thirty-four (1.9,3.3] N_3_H_1__C_4_H_3_ contacts, 281 crystal packing and 11 biological binding have not more than eight (0.5,1.9] C_3_ (S_2_)H_1__C_3_ (S_2_)H_1_ contacts. These two rules are simple. However, the second rule has 11 false negative predictions, while the first rule is much hard to interpret according to our current biological knowledge.

Finally, we would like to note that *β* contacts are all in distance-based contacts, and then those contacts in the interesting rules of *β* contacts are in fact also in distance-based contacts. But distance-based contacts have so many non-*β* contacts, masking the detection of critical specific atomic contacts in the interesting rules of *β* contacts.

#### Two misclassified examples in the decision tree of *β* contacts

**Figure 4:**
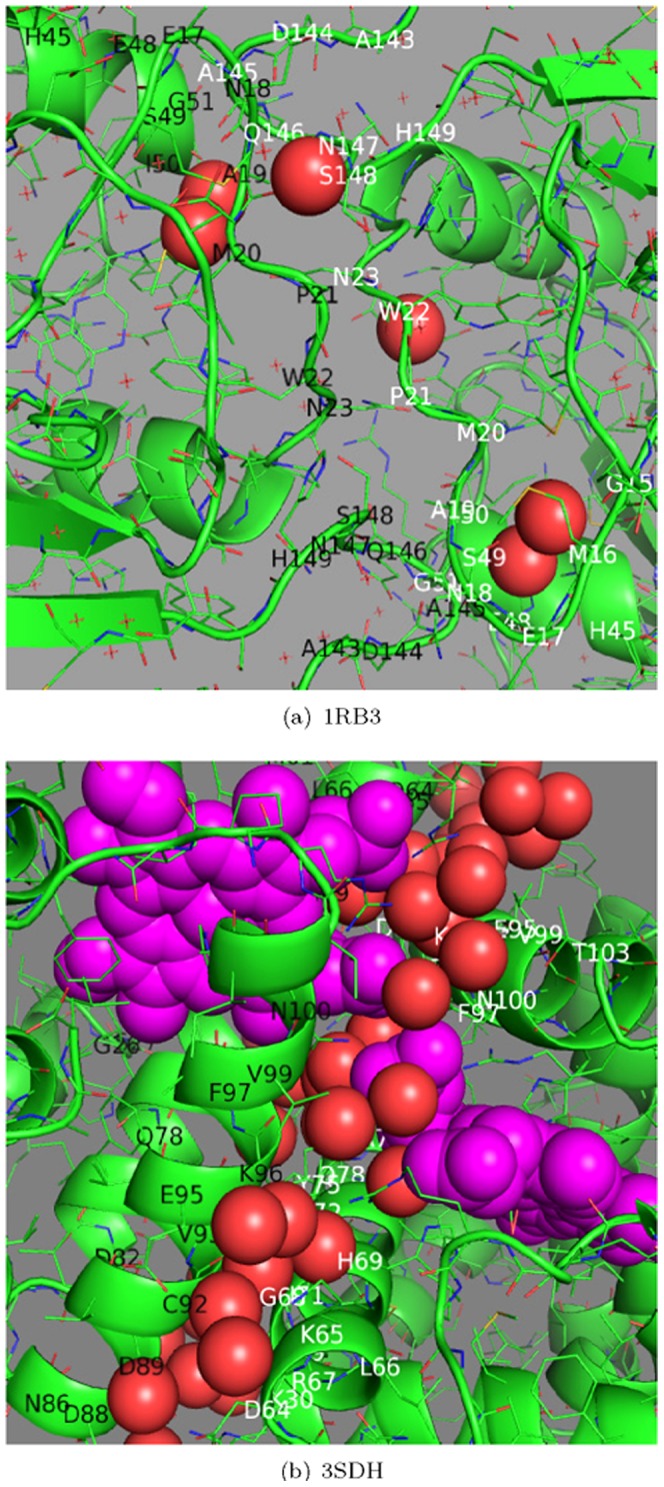
Two misclassified examples in the decision tree of *β* **contacts (better viewed in color).** (a) The crystal packing in 1RB3 is misclassified as biological binding by the biological rule R1. (b) The biological interface in 3SDH follows the crystal packing rule R2. In (a) and (b), the residues labeled in black (chain a) and white (chain b) form an interface; interfacial waters whose spatial distances to both chains are less than 3.5 Å are in the red sphere view; non-standard residues are in the magenta sphere view; Carbon: green; Oxygen: red; Nitrogen: blue.

In the decision tree of *β* contacts, each of the two interesting rules, R1 and R2, has a misclassified interface. According to R1 in the first line of Figure 3(a), 82 biological interfaces have more than four ≤0.5 C_3_ (S_2_)H_0__C_4_H_3_ contacts, and only one out of 314 crystal packing interfaces satisfies R1 in the non-redundant dataset. This misclassified crystal packing interface is 1RB3 as shown in Figure 4(a). This interface has five ≤0.5 C_3_ (S_2_)H_0__C_4_H_3_ contacts. However, there are contradictory conclusions on this interface. On one hand, the authors who determined 1RB3 in PDB recommended it as a dimeric unit; it seems that the decision tree of *β* contacts shares the conclusion with the original authors of 1RB3. On the other hand, the non-redundant dataset labels 1RB3 as ‘crystal packing’; the existing classifiers in the literature, such as NOXclass, DiMoVo and PISA, also predict it as a crystal packing. However, 1RB3's interface size is as small as 615 Å^2^, which is much smaller than the cut-off of interface ASA 856 Å^2^ suggested by the previous work [32] to distinguish crystal packing from homodimers. Meanwhile, almost all of the classifiers in the literature have the same bias–-they heavily rely on interface size. That is, 1RB3's smaller interface size can easily mislead their predictions: these classifiers are more likely to have wrong predictions for 1RB3, and tend to consider 1RB3 as a crystal packing according to the same bias of 1RB3's smaller interface size. Thus, these predictions provide nothing more than 1RB3's smaller interface size. In summary, 1RB3 may be a potential dimer, just as R1 and the original authors suggest, but whether it is actually a dimer remains a question until it can be verified in wet-lab experiments.

The second rule R2 is in the sixth line of Figure 3(a), which covers 251 crystal packing and only one biological binding in the non-redundant dataset. This biological binding is 3SDH whose interface is shown in Figure 4(b). This 3SDH interface has plenty of interfacial water molecules, and large-size non-standard residues as shown in Figure 4(b). These two kinds of molecules are not evaluated in *β* contacts so far, which may be the reason why the decision tree of *β* contacts misclassifies 3SDH.

### *β* contacts outperform previous methods in the classification of crystal contacts and homodimers

#### Feature-selection classification performance

The feature-selection classification performance of *β* atomic contacts is evaluated against those achieved by DiMoVo [11], and Block's method [28]. The best classification performance of the Block's method and the DiMoVo prediction are shown in Table 2.

In Table 2, our *β* contacts trained on the Ponstingl dataset have better classification performance than the best performance (accuracy 0.948) of the Block's method. When the Block's method uses SVM, its best accuracy is 0.919 with an RBF kernel, which is much less than our accuracy 0.959.

On the Bahadur dataset, DiMoVo's performance is recalculated by using its recalls for homodimers and crystal packing in [11]. *β* contacts have at least comparable, if not better, performance with DiMoVo. However, our *β*ACV1a and *β*ACV3 on *β* contacts adopt a linear kernel which is simpler than the RBF kernel used in DiMoVo.

#### Classification performance on the independent datasets

In addition, the classification performance of *β* atomic contacts on the independent datasets is also compared with those achieved by other methods in the literature, including DiMoVo, PISA, PITA [29], and NOXclass. Their classification results are shown in Table 3 where the performance of DiMoVo, PISA, PITA, and NOXclass on the independent datasets is recalculated by using the recall and specificity and the datasets in [11]. Here, the independent dataset has protein complexes whose proteins have less than 30% sequence similarity to those proteins of complexes in the training dataset.

Trained on the Ponstingl dataset, our *β*ACV3 of *β* contacts have accuracy of 0.937, and MCC of 0.844, which are much higher than those achieved by DiMoVo, PISA and PITA. For example, PITA achieves the best accuracy of 0.894 and MCC of 0.73 among DiMoVo, PISA and PITA; its accuracy is 4.3 percent points lower than *β*ACV3's accuracy, and its MCC is 11.4 percent points lower than *β*ACV3's MCC.

When *β*ACV1a and *β*ACV3 of *β* contacts are trained on the Bahadur dataset, they again achieve better performance than DiMoVo. Our *β*ACV3 has 0.966 accuracy, 3.7 percent points higher than DiMoVo's, and *β*ACV3 has 0.902 MCC, 12.2 percent points higher than DiMoVo's. In this case, MCC is a better metric than accuracy to compare *β* contacts with DiMoVo, since the independent-testing dataset of Bahadur is quite unbalanced: crystal packing is about four times larger than homodimers. Hence, the great improvement of MCC suggests that *β*ACV3 is much better than DiMoVo to capture protein specific binding.

When the NOXclass dataset is the training dataset, *β* contacts have much better performance than NOXclass, although *β* contacts cannot achieve better performance than DiMoVo. A reason for this is that *β* contacts can easily distinguish crystal packing from homodimers in the NOXclass dataset. Removal of several features does not change training accuracy significantly, which misleads SVM and SVM-RFE into choosing all features or fewer features as the best feature set. However, the samples in the NOXclass's independent dataset are much harder to distinguish.

In conclusion, *β* contacts demonstrate its superior classification power to the other methods in the literature under non-*β* contact definitions. This partially, if not entirely, results from the fact that the new *β* contact definition can capture specific binding patterns in homodimers and then benefit the classification of homodimers from crystal packing.

## Conclusion

The main contribution of this work is to propose the novel concept of *β* atomic contacts to identify critical specific contacts across protein binding interfaces. To evaluate the efficacy of the proposed *β* contacts, we design a new classification scheme *β*ACV for classifying crystal packing and homodimers. We compare *β*ACV's classification performance with those achieved by the existing methods on the three datasets. The promising performance achieved by *β*ACV demonstrates that *β* contacts can truly identify a compact set of critical specific contacts in protein binding interfaces which are only a small fraction of conventional distance-based contacts. Thus, *β* atomic contacts provide a new fundamental and precise unit for atomic organization in computational structural analysis. In future, *β* atomic contacts have many other applications, such as the estimation of folding and binding free energy, the prediction of binding hot spots, protein docking as well as other structural analyses for folding and binding of proteins and RNA/DNA. In these potential applications, one should pay more attention to repacking, as the exact positioning of residues is particularly important to *β* contacts.

## Supporting Information

File S1 Introduction of methods and measures, and more discussion of beta contacts.
(PDF)
