# Supplementary material for: Beta Atomic Contacts: Identifying Critical Specific Contacts in Protein Binding Interfaces
Source: PLoS One. 2013 Apr 22;8(4):e59737. doi: 10.1371/journal.pone.0059737 (PMC3632532; doi:10.1371/journal.pone.0059737)
Supplement: File S1 — Introduction of methods and measures, and more discussion of beta contacts. (PDF) [file pone.0059737.s001.pdf]

β Atomic Contacts: Identifying Critical Specific Contacts in Protein Binding Interfaces—supplementary

Qian Liu[1], Chee Keong Kwoh[1], Steven C.H. Hoi[1,*]
1 BIRC SCE, Nanyang Technological University, Singapore 639798.
∗ E-mail: liuq0011@e.ntu.edu.sg; chhoi@ntu.edu.sg

Table 1. Atomic types.

Atomic group	Chemical formula	Valency^n	Hydrogen^m	Non-hydrogen^l
N_3H_0	>N−	3	0	3
N_3H_1	>NH	3	1	2
N_3H_2	$-NH_2$	3	2	1
$N_4(_3)H_3(_2)$	$-NH^+_{4(3)}$	4(3)	3(2)	1
O_1H_0	=O	1	0	1
O_1H_0	$-O^-$	1	0	1
O_2H_1	−OH	2	1	1
$C_3(S_2)H_0$	−C<(−S−)	3(2)	0	3(2)
$C_3(S_2)H_1$	−CH−(−SH)	3(2)	1	2(1)
C_4H_1	−CH<	4	1	3
C_4H_2	$-CH_2-$	4	2	2
C_4H_3	$-CH_3$	4	3	1

^{n}: Valency of X atom;
^{m}: Hydrogen atoms bonded to X;
^{l}: Non-hydrogen atoms bonded to X.
N, H, O, C and S represent Nitrogen, Hydrogen, Oxygen, Carbon and Sulfur atoms respectively.
Most atomic groups are proposed by [1].

Introduction of machine-learning algorithms

SVM.

Support vector machine (SVM) [2] is a state-of-the-art machine-learning algorithm. It usually achieves better performance than traditional machine-learning algorithms and has been successfully applied in a wide range of applications, including in bioinformatics [3–7].

Given a binary-class dataset D, SVM constructs an optimal hyperplane based on the maximum margin principle, that is, a linear classifier $f(x) = w \cdot x - b$ where x is any input vector from D, $\cdot$ denotes dot product, and w is a normal vector perpendicular to the hyperplane. When the dataset D is not linearly separable, SVM typically uses kernel functions to map data to a high dimensional vector space and then finds an optimal hyperplane in the new space.

In this work, SVM with a linear kernel in LIBSVM [8] (the freely available SVM library) is employed to train our classifier.

RFE: a feature-section method.

Feature selection is an important pre-processing step for a classification task, since noise or irrelevant features often significantly hinder classifier construction. In β contacts, not all types of atomic pairs are

specific to protein binding; some types of atomic contacts may just happen by chance in crystal packing and homodimers. Feature-selection algorithms are able to remove noise or irrelevant features and identify relevant features in a given dataset D, which helps to construct efficient and robust classification methods. Two common feature-selection approaches are filter and wrapper. In this work, a wrapper-based approach is adopted to select best β atomic contacts.

Wrapper-based feature selection approaches employ some specific learning algorithms (e.g., SVM) to identify which features are more discriminative for classification task. However, the full space of feature subsets is often huge, i.e., 2^m by an exhaustive search where m is the number of features. Several heuristic ways are commonly used to select feature sets efficiently, including forward selection and backward elimination. Among them, SVM-RFE (SVM-Recursive Feature Elimination) [9] is one of the most successful wrappers. SVM-RFE first uses SVM learning to obtain feature weight w_i for each feature (a type of atomic contacts here). Then, it removes features (one feature in this work) with the lowest value $\|w_i\|^2$. This removal process is repeated until predefined criteria are satisfied. We implemented SVM-RFE in Algorithm 1 to find a subset of features which can produce the best empirical classification performance on datasets.

Algorithm 1 SVM-RFE in β atomic contact vectors

Input: a given data D
Output: a subset of features S_f with the best accuracy
Description:

```
set S_f = {}, and the best accuracy A_b = 0
while more than five features left in D do
    calculate classification accuracy A_c by using k-fold cross-validation
    if A_b < A_c then
        set A_b = A_c
        set S_f to all features in D
    use the linear LIBSVM to train an SVM model
    calculate weights for all features in D based on the training model
    rank the features in a descending order according to the square of their weights ||w_i||^2.
    remove the last feature from D
```

Decision Tree.

Decision tree is an easily interpretable machine-learning algorithm. It uses a recursive divide-and-conquer strategy to organize data into a tree structure. Each leaf in this tree represents a prediction by a rule from the root node to this leaf. So, given a data D, decision tree chooses a best discriminating feature to split D into several partitions. For each partition and its partitions, decision tree repeats the split until predefined criteria are satisfied. Here, we use one decision-tree implementation C4.5 [10] in Weka [11] to visualize the selected features by RFE.

Evaluation measures

The following measures are adopted to evaluate the classification performance of β atomic contact vectors:

$$\begin{aligned} recall(r.) &= \frac{TP}{TP+FN} \\ specificity(sp.) &= \frac{TN}{TN+FP} \\ accuracy(acc.) &= \frac{TP+TN}{TP+TN+FP+FN} \\ MCC &= \frac{TP\times TN-FP\times FN}{\sqrt{T\times F\times(TP+FP)\times(TN+FN)}} \end{aligned} \quad (1)$$

where homodimers are considered as the positive samples (T) while crystal packing as the negative samples (F); TP, FP, TN and FN are true positives, false positives, true negatives and false negatives respectively. So, $recall(r.)$ is the number of correct homodimer predictions divided by the number of homodimers; $specificity(sp.)$ is the fraction of correct predictions of crystal packing over all crystal packing; $accuracy(acc.)$ is the proportion of correct predictions in all homodimers and crystal packing; Matthew's correlation coefficient (MCC) is more meaningful in a dataset which has a significant imbalance between the number of positive and negative samples.

Discussion and future improvement

Discussion on Infrequent and Redundant β Atomic Contact Patterns.

In the classification of β contacts, crystal packing is used as the contrast of biological interactions. Thus, it is natural to mine those specific contacts which frequently occur in biological interactions but infrequently, if not never, occur in crystal packing. These specific contacts give us an intuitive understanding of governing principles behind protein binding. If all specific contacts can be detected, these contacts as a whole should be a perfect definition for protein binding. In this work, β contacts do suggest several biologically meaningful rules in the decision tree—those hydrophobic contacts and hydrogen bonds truly rarely occur in crystal packing. Unfortunately, the decision tree only provides a few of those specific contacts. Many of important contacts to protein binding are not detected by machine-learning algorithms here, since detection of critical specific contacts is not a trivial task.

First of all, β atomic contacts only take up to 10% of distance-based contacts. Hence, β contacts have a fairly sparse matrix D where each row represents an interface while each column denotes an atomic pair. Here, 'sparse' means D has a lot of zero values. In the sparse matrix D, rarely-occurring atomic pairs are more likely to be considered as noise features by machine-learning algorithms—noise features are generally infrequent. One good example of these infrequent atomic pairs is disulfide bonds. Disulfide bonds cost a little less free energy than covalent bonds. The occurrence of disulfide bonds is closely related to biological binding: in our non-redundant dataset with 315 crystal packing and 144 homodimers, only 1 crystal packing and 9 dimeric interfaces have disulfide bonds *(this crystal packing with disulfide bonds is removed in our analysis, because there are contradictory conclusions on whether it is a homodimer)*. So, the occurrence of disulfide bonds has high confidence to predict homodimers. However, disulfide bonds are much less frequent so that they are eliminated from the best feature set at the first several runs in SVM-RFE. This results from the facts that (i) disulfide bonds rarely occur and machine-learning algorithms, such as SVM, cannot distinguish this feature from noise features; and (ii) disulfide bonds may also be considered as redundant features. In other words, protein interactions which have disulfide bonds may also have other frequent atomic contacts, and then these interactions can be correctly distinguished by other more frequent features. Thus, disulfide bonds are not considered to be important in classification of homodimers and crystal packing. However, it does not mean disulfide bonds are not important in protein binding.

Similar to disulfide bonds, some other atomic contacts that appear frequently may also be considered as redundant features by machine-learning algorithms. The problem here is that these redundant β atomic contact patterns may be of interest yet hardly be detected. This is due to that when β atomic contact patterns are used to identify homodimers from crystal packing, it is not necessary to detect all specific atomic contacts in homodimers to obtain a good prediction; employing only several important contacts is able to produce a fairly good classifier. This is supported by Figure 3 where most crystal packing and biological binding are grouped in several sub-branches. Meanwhile, some non-specific features in binding interfaces, such as interface size, are also helpful to distinguish homodimers from crystal packing. All of these indicate that only several of specific important contacts in protein binding, not all of them, are detected in this classification. In the future, we would like to evaluate β atomic contacts in other

applications such as estimation of protein folding/binding free energy where critical specific contacts should be more important.

Discussion: Comparing β contacts to existing contact definitions

In this section, we first present various definitions proposed in previous works for the definitions of protein binding interfaces and the residue/atomic contacts, and then discuss their problems. After that, we show a real example to demonstrate the difference between β atomic contacts and the contacts under the other definitions in previous works.

Various definitions of binding interfaces and their atomic contacts: A background

One definition of residue/atomic contacts uses atomic distance [12–19]. The measure under this definition evaluates how two residues/atoms are spatially close from interacting proteins. However, there is no gold standard to define 'close' contacts between two residues/atoms. Some works consider two residues close enough to contact if the spatial distance of their CA or CB atoms, or the residue's center of mass is less than a threshold [20,21], e.g., 9 Å [13], while others take all heavy atoms of residues into account [22–24]. To define contacts between heavy atoms, two ways are commonly used in the literature [13, 20]: one is to take an absolute value, e.g., 6 Å [22], as a threshold for atomic spatial distance, while the other is to take into account the van der Waals radii of atoms and use the sum of the corresponding atomic radii plus a value as a threshold—this value can be 0.5 Å [23–26], 1.5 Å [7], 2.75 Å [27–29] or 3.0 Å [16].

A more sophisticated definition of contacts is based on Voronoi diagrams of protein complexes [6,29–33]. Given a set of points p, its general Voronoi diagram, $VD(p)$, is defined by Voronoi cells of $s \in p$; a Voronoi cell is the region comprising all points closer to an s than to any other points in p. The Delaunay triangulation of p is a graph where each node is an s and two nodes s_i and $s_j, i \neq j$, have an edge if they share a Voronoi facet. Hence, a Delaunay triangulation is the dual graph of a Voronoi diagram. In the 3D structures of protein complexes, each atom/residue can be considered as node s. Two atoms are considered to contact each other if they share a facet in their Voronoi diagram or an edge in their Delaunay triangulation.

In addition, another closely related definition of protein binding interfaces considers the change of solvent accessible surface area (ΔASA) upon the formation of protein complexes [4,14,20,34–37]. Under this definition, contact residues/atoms are those residues/atoms whose ΔASA is greater than 0.1 Å^2 [34, 36] or 1.0 Å^2 [3,4,35] upon the formation of complexes. We would like to point out that the previous two types of definitions can be used to define both interfaces and residue/atomic contacts, while the third kind of the ΔASA criterion defines binding interfaces directly.

The simple definitions above have several limitations. (i) Tsai group compared the three kinds of definitions on 592 structures of protein-protein interfaces and found that the definition based on Voronoi diagrams provides a more accurate analysis of protein interfaces than the other two criteria [21]. However, they also argued that not all of these contacts based on Voronoi diagrams have a significant energetic contribution to protein binding. (ii) The first close-distance definition is commonly used, but it only considers the spatial distance between two atoms, neglecting the local organization of their nearby atoms—whether there are other atoms between these two atoms. When the distance threshold becomes larger, the close-distance criterion will detect a huge number of contacts which are truly interrupted by other atoms. It is questionable whether these interrupted contacts have a significant contribution to protein binding. At least, these contacts should have greatly different importance to protein binding from the other "perceptually meaningful" contacts. (iii) Atomic contacts are the basic unit in protein 3D structures. If a huge number of contacts are defined without considering their local organization, it will bring a great challenge for further structural analysis of protein binding. For example, when the close-distance definition is used to define biological binding interfaces, it is still very difficult, if not impossible, to mine specific contacts

from all atomic contacts defined. Another argument is that none of these simple definitions exactly follows the biological meaning of protein interfaces. That is, crystal packing and biological binding are hard to distinguish, since these criteria define large 'binding interfaces' for a lot of false binding of crystal packing. (iv) The well-known driving forces of protein folding and binding, such as salt bridges and hydrogen bonds, suggest a completely different property from these simple definitions: they are quite "perceptually meaningful"—there are no other heavy atoms between them—instead just with close spatial distance. That is, these simple definitions above cannot exactly define hydrogen bonds and salt bridges. To the best of our knowledge, few works in the literature address the above problems of the contacts under these simple definitions.

The difference of β contacts from other contact definitions

To show the difference of β atomic contacts from distance-based and Voronoi-based contacts, Figure 2 gives an example of real contacts. The real contacts in Figure 2(a) occur between three residues in the binding site of the PDB entry 3LZF [38]. Figures 2(b)-2(d) show the distance-based, Voronoi-based, and β atomic contacts, respectively. For simplicity, in Figures 2(b)-2(d), we only consider contacts between those atoms each from the following two groups: atoms CG, OD1 and OD2 from the residue Asp52, and atoms CZ, NH1 and NH2 from Arg97. The atomic contacts between these six atoms are also shown in a 2D space.

In Figure 2(b) under the distance-based definition, the two groups of atoms are fully connected, since they are spatially close to each other. Under the Voronoi-based definition, four distance-based atomic contacts are removed as shown in Figure 2(c). These removed atomic contacts, such as the contact of CG and CZ (CG_CZ for short), are interrupted by other atoms, such as NH2 (Please refer to Figure 2(a) for detail). However, some other interrupted contacts are still detected under the Voronoi-based definition, such as the OD2_NH1 contact (*In fact, these six atoms of the two groups are almost coplanar; this indicates that OD2 and NH1 should not have enough contact center region between them; this contact is similar to the contact between i' and j' in Figure 1(d).*). In contrast, these interrupted contacts are all filtered by our β contact definition as shown in Figure 2(d). Figure 2(d) also suggests three significant contacts: the contacts between OD1 and NH2, between OD2 and NH2, and between OD1 and NH1; these three β contacts are hydrogen bonds which are obviously observed in the real contacts in Figure 2(a). Thus, β atomic contacts are also "perceptually meaningful" contacts. We believe that these "perceptually meaningful" atomic contacts can identify critical specific contacts in protein binding interfaces and aid the discovery of the principles governing protein binding.

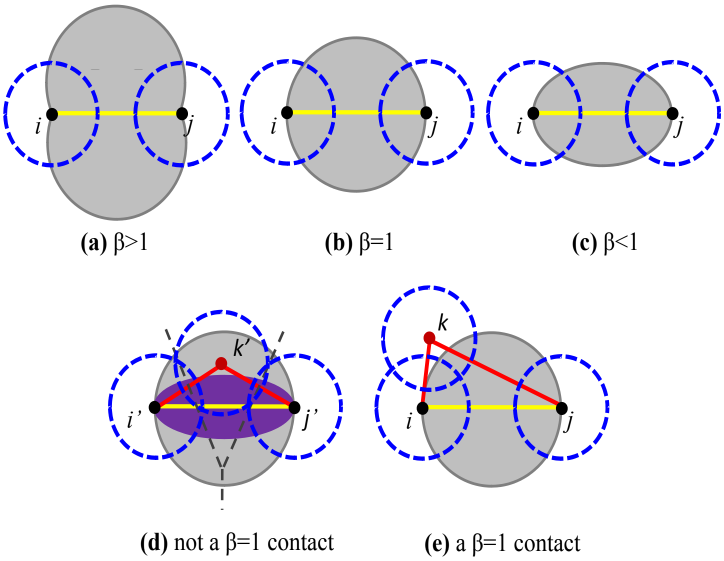

Figure 1. **β skeletons and β contacts.** Three points, i, j and k, represent the atoms. The dash circles in blue represent van der Waals spheres in 2D space. The lines in yellow are of interest. In the first row, if i and j have a β contact, the gray regions are required to contain no other atom by β skeletons when $\beta > 1$ (left), $\beta = 1$ (center) and $\beta < 1$ (right), respectively. In (d), a region in magenta is the center area which is very close around the line in yellow, and the dash lines represent Voronoi facets.

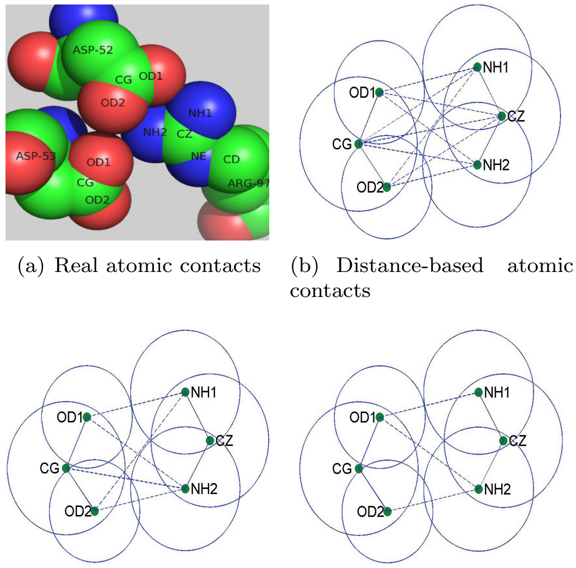

(a) Real atomic contacts (b) Distance-based atomic contacts

(c) Voronoi-based atomic contacts (d) $\beta^{=1}$ atomic contacts

Figure 2. An example of the difference of atomic contacts according to various definitions (better viewed in color). In (a), carbon, oxygen and nitrogen atoms are in green, red and blue respectively. In (a)-(d), the solid lines represent covalent bonds and the dash lines represent atomic contacts; XYZ or XY represents a specific atom of X type in the location YZ/Y in a specific residue; for example, OD1 specifies an oxygen atom in the location D1 for Asp52.

Finally, we would like to make two remarks about the definition of β atomic contacts. The first is that β atomic contacts also implicitly require a large enough angle kij or kji besides the angle requirement of ikj in β-skeletons. This implicit requirement of kij can easily be demonstrated as follows. The requirement of kji can be demonstrated similarly.

Assumptions: (i) In a β contact between two atoms i and j and their close atoms k, atoms i, j and k define a triangle as shown in Figure 1(e); (ii) k is a covalent-bond atom of i. The second assumption is needed because covalent-bond atoms have a smaller spatial distance to atom i or j than that of those non-covalent-bonds atoms. That is, covalent-bond atoms usually constrain the contacts of atom i or j in β contacts. Hence, the closest neighbor atoms k of atom i or j are generally the covalent-bond atoms of atom i or j.

Demonstration: In this triangle, the sum of the angle ikj, kij and kji is π. Then if i and j have a β contact, the angle ikj must be sharper than a right angle, that is, ikj$< \pi/2$. As a result, the sum of the angles kij and kji must be greater than $\pi/2$. Then the angle kij should be large enough, since the covalent bond of k and i generally has a much shorter length than the non-covalent contacts of i and j and of k and j in this triangle.

Although this implicit requirement on the angle kij in β contacts is not as large as that for hydrogen bonds, β contacts share an angle requirement which is required by hydrogen bonds and π-involving interactions: in a hydrogen bond, if the Nitrogen atom is considered to be k, the hydrogen atom to be i, and the Oxygen atom to be j, kij is generally required to be larger than $\pi/2$. Thus, β contacts can be considered as a generality extension of the angle requirement in hydrogen bonds and π-involving interactions. In previous

works, hydrogen bonds and π-involving interactions, which have angle requirements in contact definitions, have both been found to be important in protein binding and folding. Thus, does it mean that protein 3D structures are—instead of being a cluster of spatially close atoms with no local structures—well organized according to some rules, for example, angle requirements? The answer should be yes, but we have no idea about how atoms are well organized. In this work, β atomic contacts should be a promising attempt to detect well-organized protein 3D structures. We would like to find stronger evidence by using β atomic contacts for more applications in protein folding and binding in future.

The other remark is that some features are not directly considered in β atomic contacts, such as van der Waals forces and atomic packing density. These features may be somewhat reflected by distance-based atomic contacts if the distance threshold is large enough. In previous works, atomic packing density is observed to be an effective feature in the prediction of binding hot spots [3] and in the context of folding and protein structure prediction [39]. Therefore, these two features are advantageous in distance-based contacts for analyzing protein 3D structures. However, both of them are necessary but not sufficient conditions of protein binding—they should not be critical specific patterns of protein binding either.

Further Improvement

Despite the promising results achieved by βACV, one limitation of this vector representation is that it might not be able to discover complicated relation among atomic contacts, such as cooperativity. In fact, atomic contact graphs and their subgraphs are one of the best models of a protein tertiary/quaternary structure. An atomic contact subgraph can capture cooperativity among atomic contacts. For example, given two local structures: one has a hydrogen-bond network of hydrogen bonds between N_1 and O_1 as well as between N_1 and O_2, while the other contains two individual hydrogen bonds between N_1 and O_1 as well as between N_2 and O_2, where atoms N_1 and N_2 are hydrogen donors while atoms O_1 and O_2 are hydrogen acceptors; the hydrogen-bond network is believed to generate favorable electrostatic contribution to protein binding and strongly stabilize protein complexes [40] to a greater extent than the two individual hydrogen bonds. Furthermore, an atomic contact subgraph can also find frequent atomic contact clusters which can contribute greatly to protein binding. For instance, several contacts involving C_3H_1 clearly demonstrate a π-involving contact. More cooperative atomic contact clusters can be found in atomic contact graphs. These cooperative clusters can hardly be exactly detected in β atomic contact vectors of protein binding interfaces. But if atomic contact graphs are used to model protein 3D structures, at least two bottlenecks should be tackled in the future: first, an efficient subgraph mining algorithm is necessary to find important frequent subgraphs—they may not be connected subgraphs; and second, a much bigger dataset is compulsory, since subgraph space is very large. If the learning dataset does not have enough protein structures, subgraphs generally have lower occurrence and become meaningless.

Meanwhile, β atomic contacts of water molecules and non-standard amino acid residues are not considered in this work. This is due to the fact that crystal packing in the datasets generally contain no water information. These β atomic contact patterns will be evaluated in our future work.

References

1. Tsai J, Taylor R, Chothia C, Gerstein M (1999) The packing density in proteins: standard radii and volumes. J Mol Biol 290: 253–266.

2. Cortes C, Vapnik V (1995) Support-vector networks. Machine Learning 20: 273–297.

3. Cho KI, Kim D, Lee D (2009) A feature-based approach to modeling protein-protein interaction hot spots. Nucl Acids Res 37: 2672–2687.

4. Zhu H, Domingues FS, Sommer I, Lengauer T (2006) NOXclass: prediction of protein-protein interaction types. BMC Bioinformatics 7.

5. Block P, Paern J, Hullermeier E, Sanschagrin P, Sotriffer CA, et al. (2006) Physicochemical descriptors to discriminate protein-protein interactions in permanent and transient complexes selected by means of machine learning algorithms. Proteins 65: 607–622.

6. Bernauer J, Bahadur RPP, Rodier F, Janin J, Poupon A (2008) DiMoVo: a voronoi tessellation-based method for discriminating crystallographic and biological protein-protein interactions. Bioinformatics 24: 652–8.

7. Liu Q, Li J (2010) Propensity vectors of low-ASA residue pairs in the distinction of protein interactions. Proteins 78: 589–602.

8. Chang CC, Lin CJ (2001). LIBSVM: a library for support vector machines.

9. Guyon I, Weston J, Barnhill S, Vapnik V (2002) Gene selection for cancer classification using support vector machines. Machine Learning 46: 389–422.

10. Quinlan JR (1993) C4.5: Programs for Machine Learning (Morgan Kaufmann Series in Machine Learning). Morgan Kaufmann, 1 edition.

11. Weka Machine Learning Project. Weka. URL http://www.cs.waikato.ac.nz/˜ml/weka.

12. Ofran Y, Rost B (2003) Analysing six types of protein-protein interfaces. J Mol Biol 325: 377–387.

13. Tsai CJ, Lin SL, Wolfson HJ, Nussinov R (1996) A dataset of protein-protein interfaces generated with a sequence-order-independent comparison technique. J Mol Biol 260: 604–620.

14. Gong S, Park C, Choi H, Ko J, Jang I, et al. (2005) A protein domain interaction interface database: InterPare. BMC Bioinformatics 6: 207.

15. Lawrence MC, Colman PM (1993) Shape complementarity at protein/protein interfaces. J Mol Biol 234: 946–950.

16. Larsen TA, Olson AJ, Goodsell DS (1998) Morphology of protein-protein interfaces. Structure 6: 421–427.

17. Preissner R, Goede A, Frommel C (1998) Dictionary of interfaces in proteins (DIP). data bank of complementary molecular surface patches. J Mol Biol 280: 535–550.

18. Korkin D, Davis FP, Sali A (2005) Localization of protein-binding sites within families of proteins. Protein Sci 14: 2350–2360.

19. Davis FP, Sali A (2005) PIBASE: a comprehensive database of structurally defined protein interfaces. Bioinformatics 21: 1901–1907.

20. Glaser F, Steinberg DM, Vakser IA, Ben-Tal N (2001) Residue frequencies and pairing preferences at protein-protein interfaces. Proteins 43: 89–102.

21. Fischer TB, Holmes JB, Miller IR, Parsons JR, Tung L, et al. (2006) Assessing methods for identifying pair-wise atomic contacts across binding interfaces. J Struct Biol 153: 103–112.

22. Mintseris J, Weng Z (2003) Atomic contact vectors in protein-protein recognition. Proteins 53: 629–639.

23. Halperin I, Wolfson H, Nussinov R (2004) Protein-protein interactions: Coupling of structurally conserved residues and of hot spots across interfaces. implications for docking. Structure 12: 1027–1038.

24. Li X, Keskin O, Ma B, Nussinov R, Liang J (2004) Protein-protein interactions: hot spots and structurally conserved residues often locate in complemented pockets that pre-organized in the unbound states: implications for docking. J Mol Biol 344: 781–795.

25. Keskin O, Tsai CJ, Wolfson H, Nussinov R (2004) A new, structurally nonredundant, diverse data set of protein-protein interfaces and its implications. Protein Sci 13: 1043–1055.

26. Guney E, Tuncbag N, Keskin O, Gursoy A (2007) HotSprint: database of computational hot spots in protein interfaces. Nucl Acids Res 36: D662–D666.

27. Li J, Liu Q (2009) 'double water exclusion': a hypothesis refining the o-ring theory for the hot spots at protein interfaces. Bioinformatics 25: 743–750.

28. Liu Q, Li J (2010) Protein binding hot spots and the residue-residue pairing preference: a water exclusion perspective. BMC Bioinformatics 11: 244.

29. Li Z, Li J (2010) Geometrically centered region: A "wet" model of protein binding hot spots not excluding water molecules. Proteins 78: 3304–3316.

30. Headd JJ, Ban YEA, Brown P, Edelsbrunner H, Vaidya M, et al. (2007) Protein-protein interfaces: Properties, preferences, and projections. J Proteome Res 6: 2576–2586.

31. Cazals F, Proust F, Bahadur RP, Janin J (2006) Revisiting the Voronoi description of protein-protein interfaces. Protein Sci 15: 2082–2092.

32. Bouvier B, Grünberg R, Nilges M, Cazals F (2009) Shelling the Voronoi interface of protein-protein complexes reveals patterns of residue conservation, dynamics, and composition. Proteins 76: 677–692.

33. Poupon A (2004) Voronoi and Voronoi-related tessellations in studies of protein structure and interaction. Current Opinion in Structural Biology 14: 233–241.

34. Bahadur RP, Chakrabarti P, Rodier F, Janin J (2004) A dissection of specific and non-specific protein-protein interfaces. J Mol Biol 336: 943–955.

35. Jones S, Thornton JM (1997) Analysis of protein-protein interaction sites using surface patches. J Mol Biol 272: 121–132.

36. Bahadur RP, Chakrabarti P, Rodier F, Janin J (2003) Dissecting subunit interfaces in homodimeric proteins. Proteins 53: 708–719.

37. Chakrabarti P, Janin J (2002) Dissecting protein-protein recognition sites. Proteins 47: 334–343.

38. Xu R, Ekiert DC, Krause JC, Hai R, Crowe JE, et al. (2010) Structural Basis of Preexisting Immunity to the 2009 H1N1 Pandemic Influenza Virus. Science 328: 357-360.

39. Day R, Joo H, Chavan AC, Lennox KP, Chen YA, et al. (2013) Understanding the general packing rearrangements required for successful template based modeling of protein structure from a CASP experiment. Computational Biology and Chemistry 42: 40-48.

40. Sheinerman FB, Honig B (2002) On the role of electrostatic interactions in the design of protein-protein interfaces. J Mol Biol 318: 161-177.
